# Mr. Mantal, in Reply to Dr. Kinglake, on Gout

**Published:** 1805-12

**Authors:** James Mantal

**Affiliations:** Bromley, High Elms


### Mr. Mantal, in Reply to Dr. Kinglake, on Gout.

[ Continued from our last, pp. 355—361. ]

This mode of reasoning, if it be just, applied to Dr. Kinglake's argument, by which he would prove gout to be a disease of the ligaments and tendons, from a constitutional sympathy, resembling that which occurs in sprain, must deprive it of all authority. His argument is a paralogism, which, by proving too much, really proves nothing. It would assimilate gout not only to sprain, but to all those disorders and accidents, in which a similarity of systematic commotion is discoverable. Until, therefore, Dr. Kinglake can prove by less controvertible arguments, until he can appeal to *facts* for the truth of his assumption, I must still remain unconvinced that gout originates and chiefly continues on the tendons and ligaments; and *agree rather in opinion* with those Physicians, who describe it, as an "inflammation of the *membranes that cover the joints*." Dr. Kinglake asks me, *what are those membranes?* denies the existence of any, excepting the cellular; and affirms, that even the cellular no where distinctly envelopes the joints. It might be sufficient for me to refer him to those for an answer, who first taught that doctrine. I only declared my assent to their opinion. I did not claim to be its author. But having adopted their term, I am ready to declare the sense in which, I conceive, they employed it. I had said, that the actions of the inflamed *membranes of the extremities* were associated with those of the *membranous parts* of the stomach and liver. If Dr. Kinglake had duly enquired, what kind of analogous structure equally subsists in those different parts of the body, his question had been superfluous. It is evident, that membrane is here used in a sense, different from that in which Dr. Kinglake would employ the term. Not only the whole surface of the body and all its cavities are covered with membrane, but it forms every cell, lines every vessel, unites every fibre; even that complicate intertexture, composed of the mouths of the absorbent system, the excretory ducts of the capillaries, and their attendant arteries, veins and nerves, must be, strictly speaking, all membranous. (See Darwin, Sect. 26). The minuteness of these vessels can be no objection to this definition; for their sides must consist of the same simple material as the more perceptible membranes, and are, therefore, equally entitled to the same denomination. The immediate seat of inflammation in gout, I suppose to be; the capillary vessels, or glands; and that they secrete, in their *inflamed state,* that viscid humour, which hardens into chalk-stones, in consequence of the absorption or evaporation of the more fluid part. The same system of vessels, when inflamed, probably effuses into the interstitial parts of the liver, that solid material called *scirrhus;* when the association between that organ and the stomach and skin, is prevented from taking place by unknown causes, and no gout is produced on the membranes of the joints. But whether it is this system of vessels only, or the whole of that intricate construction of absorbents, exhalants, arteries, veins, and nerves, by which the inflammation is propagated from the viscera to the joints; and whether the tendons and ligaments might not from sympathy of contiguity fall into the general inflammation of the surrounding parts, it would perhaps be of no use to enquire. Science would gain nothing by so minute an investigation. It is a sensible remark of some writer, whose name I do not recollect, that "Mankind is always more benefited by attending to the *large, open, and perceptible* parts of our frame, than by studying too much those finer nerves and vessels, the conformations and uses of which will for ever escape our penetration."

Dr. Kinglake thinks the doctrine inadmissible, which represents torpor as a cause of inflammation. But I appeal to facts. The torpor of an intermittent occasions the succeeding hot stage. The torpor of frozen hands is the cause of their subsequent inflammation. In affirming torpor to be the cause of inflammation, I used a popular, not a philosophical language. I diligently avoided the disputable question, in what manner it produced that effect; but in a thousand instances the *fact* is open to observation. Darwin's theory of the accumulation of vital power during the inactivity of the torpid parts may be true or false; I had nothing to do with it. The *fact* alone was the foundation of my reasoning. The theory seems to be needlessly introduced by Dr. Kinglake, for the purpose of refuting it. It is founded upon what is indisputably true, that vital power, by whatever name it is called, is the only efficient cause of motion or action to animated matter. Philosophically speaking, torpor is but a previous condition, the *inert* cause; the vital power, the only *efficient* cause of inflammation. It is inconceivable to me, but it may not be so to Dr. Kinglake, how torpid parts can act with increased energy or become inflamed, the stimulus not.

not being greater than usual, unless we admit the explanation, of the *accumulated vital power* during their quiescent state. Alternate rest and activity is the very law of our Nature; thus the quiescence of night recruits our muscles for the labour of the ensuing day; in other language, "the vital power accumulates in circumstances of inaction." But how does Dr. Kinglake attempt to refute this doctrine? "If a part be torpid, he says, it must be deficient in the necessary energy to generate an excess of vital power." Here is a petitio principii, which will never be granted; an assumption, which rests upon the foundation of no proof; it is determining, what will probably for ever escape our knowledge, *where the vital power is generated.* Darwin, however, at whose doctrine this shaft is hurled, would never suppose that it was generated in the torpid parts of the system; or that they, in any state, possessed an energy, independently of the vital power. Is not the vital power *dissipated* by the activity of the animal motions? But how can it be *dissipated* and *generated* by the same activity? This is, indeed, blowing hot and cold at the same time; it is the "*inflammatory torpor*" of Dr. Kinglake's own imagination; the offspring perishes, before it comes to the birth; nay, before it is *generated* in the nidus, Dr. Kinglake has provided for it. He first supposes, that a *torpid* part cannot *generate* vital power. What is this but declaring that its *activity* is necessary to the production of that source of life? And then, "when vital power abounds, that it is *dissipated* by the irregular actions of violent disease." This argument, with respect to *consistency*, seems allied to those, which, as Dr. Kinglake observes, "may be convenient to hypothetical reasoning, but will not satisfy the just demands of scientific enquiry."

Another objection, urged by Dr. Kinglake, convinces me, that he did not accurately conceive either my meaning, or the theory, which he opposes; when he thought the following interrogatories, in the remotest degree, applicable to its refutation. He asks, "how does the idea of torpor comport with the usually existing state of the system of those, who are most afflicted with gout? Does not an attack commonly ensue a series of intemperance in eating and drinking, or some other stimulant excess? Does it arise after a course of abstemiousness? Is it not manifoldly proved, that the torpor (if Mr. Mantal will have it) of extreme sobriety and temperance very generally prevents the periodical recurrence of gout?" The proper answer to these questions will direct us to a conclu-

 sion, sion, very different from that which Dr. Kinglake would deduce from them. The torpor of the vital organs I suppose to be the consequence of excess; the permanent result not of occasional debauches, but of a long course of habitual, excessive, previous stimulation; which first produces an increase of all the animal motions. But debility, or inirritability, with torpor, is the invariable consequence of all undue action; whether we apply the principle to the muscles, that are obedient to the will, after violent and long continued exertion; or to the eyes, after exposure to too strong and intense a light; or to the stomach and liver, that have been subjected to the constant influence of excessive *vinous* stimulation.

The *natural* stimulus of the stomach, that which excites it into sufficiently energetic action without inducing premature debility of that organ, is evidently mild animal and vegetable substances. That these are the only proper food of man, to the exclusion of the more stimulant kinds, as of spirits, wine, and spices, is rendered probable by the consideration, that their elements are found by chemical tests to be the same, and almost to exactness in the *same proportion to each other*; a most material circumstance, as they are found in our own substance. But in wine, for instance, by far the most common and most legitimate parent of gout, the quantity of hydrogen far exceeds the proportion of the other elements; and possessing great stimulant powers, excites the parts to which it is applied, into unnatural and excessive action, to be followed by a proportionate degree of debility and consequent torpor.

If the stomach is the part to which this unnatural stimulus is applied, whence does it happen that the morbid impression, as in gout, discovers itself in the extremities? The vis medicatrix naturæ may produce a metastasis of the morbid action from the vital to the most remote parts. But possibly some may consider this as an imaginary power. I will at present wave the question of its existence, and try to explain the fact another way. I bow to the authority which ordains, "Nec Deus intersit, nisi dignus vindice nodus inciderit." The explanation, indeed, is beset with difficulties; it is a "nodus," but perhaps not "Deo vindice dignus;" it may be untied without supernatural aid.

It is evident that the stomach of those most afflicted with the gout has been unduly, and disproportionately to the other parts of the system, excited into action for a long long course of years by stimuli stronger than natural: whence an increase of action and of strength at first, and subsequent debility of that organ, the invariable result. Other parts of the system are excited into action by their own peculiar stimuli; which perhaps are always the same, or nearly so; for instance, the sanguiferous by that of blood, the lacteal and lymphatic by chyle and lymph; which are the natural and appropriate stimuli of their respective systems of vessels; exciting them into sufficiently energetic contractions without inducing subsequent debility. When the contractions of the stomach are stronger than natural, owing to excessive stimulation, as of wine, other parts are stimulated not only by their own peculiar fluids, but by sympathy and association with the increased actions of the stomach. It is probable that those actions, which are the result of associations, are in general performed with greater energy than the primary ones, which occasioned them. This question is, perhaps, important; but I will not now enter upon it; though the solution of, it would probably shed considerable light on the production and remote causes of many diseases. Thus the liver is confessedly more hurt than the stomach, although the stimulus is only applied to the latter organ. A *triple* association may here be supposed to take place; first, between the stomach and the duodenum; then between, the duodenum and the ductus communis; and, finally, between the ductus communis and the secretory vessels of the liver. Dissections make it highly probable, that the injury is conveyed to the liver through the means of the ductus communis. Dr. Saunders (page 240 of the last edition of his excellent Treatise) tells us, that "the diseased structure may be traced from the stomach along the course of the ductus communis, and that he has frequently seen these ducts so contracted and thickened, that they could not transmit bile."

The purport of this reasoning will now be obvious, and will furnish us with a plausible, if not a real, explanation, why gout should particularly exert its influence on the extremities. The cutaneous capillaries had been excited into undue actions by their association with those of the stomach, or liver, or both; these actions are performed with greater energy than those of the stomach or liver, because they are *actions of association*. The consequence of their greater activity must be the same, which we find to result invariably from all unnatural exertion; proportionate debility and torpor. And the reason why these effects should appear at the extremities rather than in other parts may be, that the increased action is propagated from the central to the extreme parts, and acquires strength in its progress; whence greater activity at the joints, and greater consequent debility and torpor. The effect of this torpor, the peculiar characteristic inflammation of gout.

Much of this reasoning, it must be owned, respecting the *manner* in which gout is produced, is conversant on a subject of great obscurity; and therefore will probably be deemed fanciful in the judgement of the most candid critics. But whether it be founded in truth or error, is of little consequence, provided the *fact* be allowed, which I principally contend for, and which, I think, is undeniable, that *the general torpor of the system, for a few days or a week, is the usual precursor of a gouty attack.* For every unprejudiced mind will be convinced, that this torpor is in some manner intimately connected with the subsequent inflammation; and that the *constitutional* ailment deserves our principal regard, when we institute a mode of cure for the *local disease.* Dr. Kinglake speaks with approbation of my comments on Mr. Baker's case; but the point chiefly laboured in them, which Dr. Kinglake himself notices, is, that "*systematic tone* may be established through the efficiency of *articular inflammation.*" It is far from being certain that the patient will *not,* it is highly probable that he *will,* if the inflammation be cured previously to the establishment of systematic tone, be liable to worse disorders, equally the consequence of the defect of systematic energy. Gout seems to be the commencement of disorders originating in a broken constitution, in a decay of those essential vital organs, on the due performance of whose functions the energy of the inferior parts in a great measure depends. Hence palsies, apoplexies, dropsies, spasms, wandering pains, tremblings, sickness, vertigoes, loathing of food, and a hundred other disorders, which are *usually imputed to the gout;* but their true cause, I believe, is rather to be sought in the *decay of the vital organs,* their previous excessive stimulation, than their unsusceptibility of being duly stimulated, and their consequent debility and torpor. The same disorders may be expected to arise, with still greater certainty and with aggravated symptoms, if the inflammation of the extremities be encouraged, or suffered to rage, beyond what is necessary to energize the system as far as it is capable of energy, and restore the sufferer to his usual state of imperfect health. For then, to all the other causes of decay, the debilitating effects of protracted protracted pain and violent excitement, arising from the inflammation, are to be added.

Notwithstanding the length to which these remarks have been carried, it is impossible for me to conclude without requesting a little further indulgence. In commenting on Mr. Mace's case, I had taken no notice of a circumstance mentioned by the Reporters, Messrs. Scott and Taynton, that "the patient had on former occasions suffered as much constitutional indisposition when the parts affected were covered with flannel, as under the present cooling mode of treatment." Dr. Kinglake says, "the entire silence with which this important fact is passed over, presents a regretful instance of Mr. Mantal's discovering *more solicitude to confirm his own speculation, than fairly to solve the question at issue.*" The motive, here imputed to me, is totally inconsistent with *the love of truth.* The wilful concealment of important facts is just as criminal in my eyes, as *the forgery of cases* can be in the estimation of Dr. Kinglake, for whatever purpose either of those crimes is committed; and for the same reason, because it is equally injurious to truth. Dr. Kinglake, therefore, in accusing me of this crime, condescends to imitate Mr. Edlin, against whom a general outcry has been raised, because he only intimated to his dying friend, at a moment of extreme anxiety, when reflection was asleep, that possibly Dr. Kinglake's cases were coined for the purpose of establishing his theory. But I am here taxed with the concealment of important facts, when no friend is to be saved by the innocent fraud, when reflection is perfectly awake, and when it is evident that nothing could be more senseless than to attempt a concealment of what had been openly published to all the world. I hope Dr. Kinglake will believe me, when I assure him that nothing but truth is the object of my research, and of all my speculations; and that he will certainly at all times be mistaken, whenever he shall attribute motives to me different from those which I profess, or that are incompatible with the strictest honour and sincerity.

I will now give him my reason for passing over in silence what he is pleased to call "*an important fact.*" Had the cold not been employed, I am ready to admit the extreme probability that this patient would have suffered constitutional ailment as at former times. But perhaps the immediate cause would not have been so conspicuous as it was on this occasion. Habit alone might have produced it, at least must have created a predisposition to it so strong,

 that that the smallest debilitating influence would certainly have occasioned similar systematic ailment to appear. This concession ought not in my opinion to lessen any one's belief, that the cold was that debilitating influence, which operated the systematic disorder at this time. We are told, "No sooner was the first wet cloth applied, (and it was unremittingly applied for two hours) than an unusual torpor of the system, with sickness and pain of the stomach, was the immediate consequence." Who, but Dr. Kinglake, can doubt, whether the cold, or previous habit, was immediately concerned in producing these effects? My conviction was so strong, that the mischief resulted from the cold, that I intentionally overlooked (but not with *that* intention which Dr. Kinglake attributes to me) an observation, to which I thought little regard was due; not only because it seemed to unsay all that had been said before, (for the Reporters evidently intended to convey the notion, that the mischief resulted at this time from the cold,) but because the observation is expressed in *general* terms; because no particulars of the attacks alluded to are detailed, from which to form a correct judgement; and because I was commenting, not on the former fits of gout with which the patient had been attacked, but only on that, with the particulars of which I was made acquainted The Reporters would have been more consistent either in suppressing the remark entirely, which in its present situation can only tend to throw all into confusion, and to invalidate, if not totally to destroy the inference which was intended to be drawn from the statement just before given; or else, along with the remark, in *detailing the particulars* of one or more of those former attacks, for the purpose of enabling us to discover, if possible, what other debilitating influences might have occasioned the same constitutional effects, notwithstanding the flannel over the parts affected.

In respect to the unfortunate case communicated by Mr. O'Neal, though nothing can be more evident than that the theory is not responsible for the *whole* nor for the *greater part* of the mischief which ensued, yet I cannot entirely absolve it from some share of blame in the first instance. Was there not "*excessive temperature*" in the considerably *painful and inflamed feet*? And when *that* is either "threatened by uneasiness, or characteristically shown by violent inflammation and pain," we are directed to "resist it by the topical use of cold water." We are told that this treatment, on the principles of the theory, "is

"is replete with caution." It is granted; but the principles of the theory in excluding the viscera from gout are denied. The internal affection in this case was of the utmost consequence; but the Theory is silent respecting it, the *excessive temperature* of the part is the only criterion. Dr. Kinglake will not contend for a torpor of the viscera in this case, while the foot was considerably painful and inflamed: For he assures us, that "as the viscera are highly susceptible of original as well as sympathetic inflammation, and as it is not likely that gout should inflame the dense structure of the joints and fail to produce inflammatory effects on the vital organs, so it is at variance with his uniform observation that visceral excitement in gout should be founded in *torpor*." And yet both Cullen (First Lines, 524) and Heberden (p. 44, Comm.) never met with an instance of gouty *inflammation* attacking the viscera. Here is direct evidence adduced, both in affirmation and denial of a matter of fact. It is but reasonable that judgement should go with the majority of witnesses.

I am aware that Dr. Kinglake's Theory does not admit of inflammatory gout attacking the viscera (p. 41 and 131 of the Dissertation), and that in this respect it accords with the experience and observation of those great men, whose authority is thus set in opposition to him. But neither will he admit of the torpor; that is, there is neither excess nor defect of action in the constitutional ailment, which so frequently occurs before and during the paroxysms. But where there is neither excess nor defect of action, there must be perfect health; an opinion, which, as applied to the affection of the system in gout, will hardly obtain a *plurality* of suffrages in its favour.

I have now completed my design. I am not conscious of *passing over in silence* any arguments or facts, that can be deemed important towards elucidating the question I wish to solve. Truth is as congenial to my mind, as it can be to Dr. Kinglake's. But it is the lot, and the misfortune of humanity, to be much oftener in error than in the right. There is more of uncertainty than of certainty in all our reasoning; there are, indeed, but a few certain truths. Convinced of this, if I am unable to overcome my opponent by dispassionate argument, I will disdain to have recourse to the aid of invective. If I can give him "implicit credit for integrity and honest intention," I will spare him the reproach of "intemperate zeal." Dr. Kinglake knows that if his theory be founded in truth, it has nothing to fear fear from any one's "*declamatory refusal.*" Like pure gold, it will come out of the furnace unchanged in its properties. It is the interest of the public, that it should be put to the severest test; and no attempts that are conducted with moderation, that tend to ascertain and determine its validity, its claim to the high character of truth, can properly be deemed "premature."

I am, &c.

*Bromley, High Elms, Oct.* 13, 1805. JAMES MANTAL.

